# Mating experiences with the same partner enhanced mating activities of naïve male medaka fish

**DOI:** 10.1038/s41598-022-23871-w

**Published:** 2022-11-16

**Authors:** Masahiro Daimon, Takafumi Katsumura, Hirotaka Sakamoto, Satoshi Ansai, Hideaki Takeuchi

**Affiliations:** 1 Graduate School of Life Sciences, Tohoku University, Sendai, Miyagi 980-8577 Japan; 2 Graduate School of Natural Science and Technology, Okayama University, Okayama, Okayama 700-0082 Japan; 3 Department of Anatomy, Kitasato University School of Medicine, Sagamihara, Kanagawa 252-0374 Japan

**Keywords:** Zoology, Animal behaviour

## Abstract

Mating experience shapes male mating behavior across species, from insects, fish, and birds, to rodents. Here, we investigated the effect of multiple mating experiences on male mating behavior in “naïve” (defined as sexually inexperienced) male medaka fish. The latency to mate with the same female partner significantly decreased after the second encounter, whereas when the partner was changed, the latency to mate was not decreased. These findings suggest that mating experiences enhanced the mating activity of naïve males for the familiar female, but not for an unfamiliar female. In contrast, the mating experiences of “experienced” (defined as those having mated > 7 times) males with the same partner did not influence their latency to mate. Furthermore, we identified 10 highly and differentially expressed genes in the brains of the naïve males after the mating experience and revealed 3 genes that are required for a functional cascade of the thyroid hormone system. Together, these findings suggest that the mating experience of naïve male medaka fish influences their mating behaviors, with neural changes triggered by thyroid hormone activation in the brain.

## Introduction

The roles of mating and social experience in shaping male mating behaviors have been compared across species since the 1950’s^1^. Many similarities and differences exist among species in the effects of mating experiences on the motivational and performance aspects of mating behaviors^2–5^. For example, in fruit flies (*Drosophila melanogaster*), the first mating experience in males shortens the latency to the first courtship with a female^2^. Furthermore, in a competitive mating situation between 1 female, a naïve male, and an experienced male, the experienced males more frequently exhibit abdominal bends (attempted copulation) than naïve males. In rodents such as mice (*Mus musculus*)^3,4^ and rats (*Rattus norvegicus*)^5^, the first male mating experience decreases the latency to the first intromission, i.e., mounting behavior with penis insertion. In fish species such as the blue gouramis (*Trichogaster trichopterus*), similar effects of repeated mating experiences are also observed^6^. In birds such as Japanese quail (*Coturnix japonica*)^7^ and ring doves (*Streptopelia risorii*)^8^, naïve males usually approach the females, but some of them fail to copulate on their first encounter. By their second encounter, however, most males successfully complete copulation behaviors in a species-specific manner^7,8^. In addition, mating experience activates mate preference in mosquitofish (*Gambusia holbrooki)*; Vega-Trejo et al. quantified mate preference in mosquitofish using a 3-chamber test, and demonstrated that the amount of time spent with a novel female was significantly increased by the mating experience, but not by visual and olfactory familiarization^9^.

In rodents, the behavioral change triggered by the first mating experience is associated with changes in the neural circuits and substrates in the brain. The number of neurons in the olfactory bulbs and the density of mushroom spines in the medial preoptic area (mPOA) are increased by the first mating experience in mice^4,10^. In contrast, the mushroom spine density in the mPOA decreases and the expression of *vgf,* which encodes the neuropeptide precursor VGF in the mPOA, affects behavior (shortening mating latency) following mating and ejaculation experience in male rats^5,11^. We recently reported that the expression of gastrin-releasing peptide and oxytocin receptors is increased in the spinal ejaculation generator in the lumbosacral cord after the first mating experience with ejaculation in male rats^12^. Furthermore, the first mating experience also decreases neuronal activity in the center part of the mPOA, suggesting that the first mating experience reconstructs the neural network associated with male mating behavior^13^. No studies to date have revealed the neural/molecular mechanisms underlying behavioral changes dependent on the mating experience in fish species.

To address this question, we used medaka fish (*Oryzias latipes*) in the present study. There are many advantages to using medaka fish for studying mating behavior. First, medaka mating behavior comprises several steps (approach, courtship display, wrapping and spawning), which allows for quantification of male mating activity under laboratory conditions^14^. Second, as the female reproductive cycle is 24 h, and therefore the same female ready to spawn can be used for mating tests every morning^15^. Third, medaka is a model animal for molecular genetics, and state-of-the-art molecular genetic techniques are available. Here, we show that mating experiences with the same partner enhanced mating activities of naïve males, but not of experienced males who had mated more than 7 times. Furthermore, transcriptome studies suggested the possible involvement of the thyroid system in the behavioral changes dependent on the male mating experience in medaka fish.

## Methods

### Ethics statement

All the methods in this study were carried out in accordance with relevant guidelines and regulations. The work in this paper was conducted using protocols specifically approved by the Animal Care and Use Committee of Okayama University (Permit Number: OKU-2015467) and Tohoku University (Permit Number: 2022LsA-003). Surgical dissection of the brain to extract total RNA was performed under deep anesthesia using ice, and all efforts were made to minimize suffering following the NIH Guide for the Care and Use of Laboratory Animals Fish and breeding conditions. The study was carried out in compliance with the ARRIVE guidelines (https://arriveguidelines.org/arrive-guidelines).

### Animal maintenance

All fish (*Oryzias latipes*; d-rR strain) were bred in our laboratory. Fish larvae were fed Paramecium or small pellet foods (Hikari lab., Meito system, Japan or Medaka no Mai Next, Kyorin, Japan), juveniles were fed small pellet foods (Medaka no Mai Next), and adult medaka were fed pellet foods (TetraMin, Tetra, Germany or Otohime B2, Marubeni Nisshin Feed, Japan) a few times a day. Juvenile and adult fish were fed brine shrimp once a day. Medaka were maintained in groups in plastic aquariums (13 × 19 × 12 cm height) or polypropylene containers (48 × 36 × 20 cm height). The water temperature was maintained at 24–28 °C with white LED lights (Eco-slim, OHM ELECTRIC INC, Japan) for 14 h per day (08:00–22:00).

### Animal preparation for mating tests

Adult male (> 5 months of age) and female (> 3 months of age) fish were used for this experiment. To prepare “naïve males”, we separated sexually immature males from females 1–2 months after hatching. We determined their sexes based on the body color difference^16^ and fin shape. We used sexually matured females that had spawned fertilized eggs continuously for at least 3 days as sexually matured ready-to-spawn eggs. In the present study, we defined “naïve” males as sexually inexperienced and “experienced males” as adult males that had mated with females more than 7 times.

### Mating test using fixed dyads

The mating test was performed as previously described^17^. To separate the male from the female, a plastic cup (CE-300, Kenis, Japan, 37 mm [radius] × 90 mm [height]) with white opaque paper was used from the night to the next morning (16:00–10:00). The day before the mating test, the opaque plastic cup with the naïve or experienced male was placed into a tank containing a female. The next morning, a male was released into the tank containing the female, which allowed them to begin their mating behavior (9:30–10:30). We recorded their mating behavior for 15 min using a Web camera (BSW200MKB, Buffalo, Japan). We repeated the mating tests for 7 days (times) using the same dyads. If a female did not spawn at least once over the 7 days, we excluded all data from the analysis. We manually measured the timing of the courtship display (male quick-circle dance), wrapping (crossing each body), wrapping rejection (wrapping with no spawning), and spawning by viewing the video, and calculated the latency to mate (period from releasing the male to the wrapping with spawning), the latency to the first courtship display, and the latency to mate after the first courtship. Transition probabilities (courtship from courtship [c—> c], wrapping from courtship [c—> w], courtship from wrapping rejection [wr—> c], and wrapping rejection from wrapping [w—> wr]) were calculated by dividing the number of each behavioral transition by the total number of transitions. Experienced males who had mated with females more than 7 times were used for the same experiments as a control for 7 or 3 days (times).

### Mating test using swapped dyads

To determine whether the mating experience with the same partner was essential for changing the mating behavior, we performed the mating test using fixed and swapped dyads using naïve males continuously for 3 days (times). The procedure was the same as for the mating test described above.

### Statistical analysis

Statistical analysis was run by R (version 4.0.5) with generalized linear mixed models (GLMMs) by the “glmer” function in the package lme4 (version 1.1–27) to reveal whether the number of matings (experience) affected medaka mating behavior. The gamma distribution (latency to mate, latency to the first courtship display, and latency to mate from the first courtship) and Poisson distribution (numbers of each event; courtship displays, wrappings, wrapping rejections, and transition probabilities of each behavioral transition; courtship from courtship [c→ c], wrapping from courtship [c→ w], courtship from wrapping rejection [wr→ c], and wrapping rejection from wrapping [w→ wr]) with a log link function were used for each statistical analysis. Transition probabilities were analyzed using the offset function in lme4 (e.g., model = event numbers ~ mating times + type (naïve or experienced) + type : mating times + (1|experimental No.) + (1|male) + offset(numbers of total events). Experimental No. refers to the laboratory in which the behavioral experiments were performed and individual male and female (only in swapped dyads) identification numbers of males for the fixed test and females for the swapped test were included as random intercepts (e.g., model = latency ~ mating times + type + type : mating times + (1|experimental No.) + (1|male)). We constructed both full models including mating times as an explanatory variable and null models with no explanatory variable, and then compared the models using the likelihood ratio test. To select the model with the best predictability, we compared the Akaike Information Criterion (AIC) between the 2 models. When the likelihood ratio test indicated a significant effect on the mating times (*P* < 0.05), adjusted *P* values calculated using the emmeans package (version 1.6.1) with the Tukey method are shown for post hoc test.

### RNA-Seq and data analysis

Whole brains with the pituitary were collected from naïve males with no mating experience and post-naïve males (after 2 mating experiences) in the morning just before the third mating. The dissected brains were stabilized with RNA-later (Thermo Fisher Scientific, USA) until the extraction steps. Total RNAs were extracted using TRI Reagent (Cosmo Bio, Japan) and then purified using an RNeasy plus mini kit (Qiagen, Germany). RNA extracts with the same concentrations from 3 individuals were pooled together to form 1 RNA-seq sample. There were 2 naïve and 2 post-naïve samples (i.e., 4 total samples). All library preparations and sequencing were outsourced to a company (GENEWIZ, Japan). The cDNA libraries were prepared using a NEBNext Ultra RNA Library Prep Kit for Illumina (New England Biolabs, USA). The libraries were multiplexed and loaded on an Illumina HiSeq X Ten (2 × 150 bp, Illumina, USA). The reads were trimmed using Cutadapt^18^ (v1.9.1) and then mapped to medaka reference genome sequences (ASM223467v1) with gene models in NCBI RefSeq (Annotation Release 103) using HISAT2^19^ (v2.0.1). We used edgeR^20^ (v3.4.6) for differential expression analysis. After normalization with the TMM method, differentially expressed genes were identified with an FDR adjusted *P*-value < 0.01 and |Fold change|> 2 by Fisher’s exact test.

## Results

### Mating experience of naïve males decreased the latency to mate with the same partner

**Figure 1:**
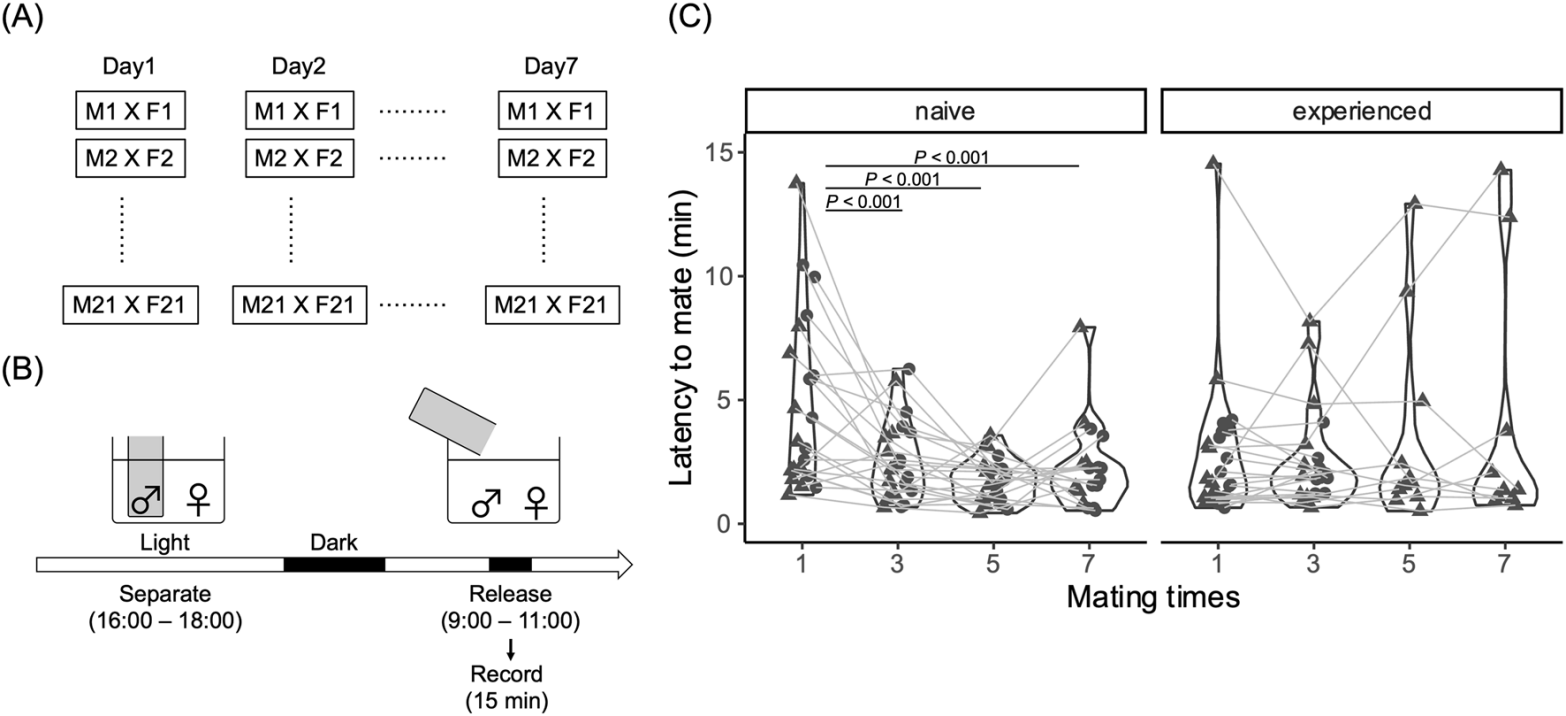
Design and results of the mating test using naïve males. (**A**) (**B**) Outline of the behavioral experiment. (**A**) Twenty-one (naïve) and 23 (experienced) males were used for the study. Male and female dyads were fixed during the experiment and the experiment was carried out for 7 consecutive days. (**B**) Experimental procedure. The male was separated a day before the experiment and released to the female the next morning. Their mating behavior was recorded for 15 min and analyzed. (**C**) Behavioral test results. Left and right panels show the transition of the latency to mate in naïve and experienced males, respectively. The latency significantly decreased in naïve males depending on the number of matings for 7 days, but not in experienced males. Each dot represents the results of each individual and the shapes show the experimental No (carried out 2 laboratories). *P*-value shows the results of the post hoc test with Tukey’s adjustment method in a generalized linear mixed model (gamma distribution, log link function).

To investigate how the mating experience of naïve males could influence mating behavior in medaka fish, we performed mating tests using naïve males and experienced males that had mated with females more than 7 times. To prepare naïve males, we separated juvenile males into groups and bred them without any females until performing the mating tests. We also prepared sexually mature females (> 3 months after hatching) for the mating test. The mating tests were carried out using 21 (naïve males) and 23 (experienced males) fixed dyads as a control for 7 days (7 times) (Fig. 1A,B). Next, we used experienced males that had obtained the previous mating experience with the same female more than 7 times. To compare the experienced males with the naïve males paired with novel partners, we changed the female partners of the experienced males just before the mating tests (Fig. 1C). The latency to mate was defined as the interval from “releasing the male” to “crossing with spawning”. To evaluate the transition of the latency to mate between naïve and experienced males, we used a GLMM (AIC (full) = 1758.40, AIC (null) = 1790.37, deviance = 1736.40, deviance (null) = 1782.37, Chisq = 45.98, Df = 7, Pr (> Chisq) = 8.84E−08), and the results of the post-hoc test revealed that the latency significantly decreased after the first mating experience and biased the distribution for 7 days only in naïve males, and not in experienced males (Fig. 1C, Table S1). Together, these findings indicated that the male mating experience influenced medaka mating behavior only in naïve males. Therefore, we concluded that the mating experience of naïve males decreased the latency to mate with the same partner.

### Mating experience of naïve males enhanced both male and female mating activities

**Figure 2:**
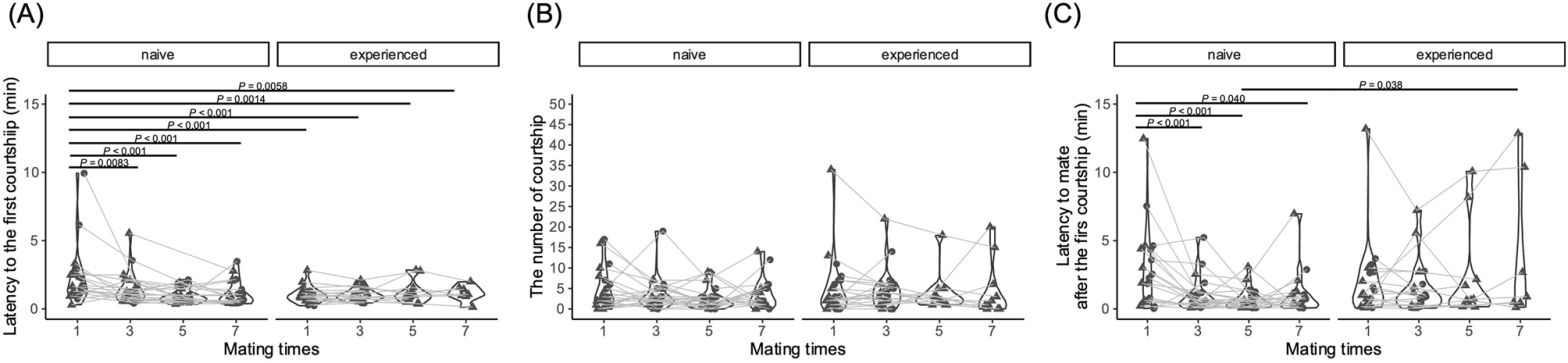
Change in the courtship behavior, latency to the first courtship display (**A**), and the number of courtship displays (**B**) and the latency to mate from the first courtship display (**C**). Each dot represents the results of each individual and the shapes show the experimental No (carried out 2 laboratories). (**B**) *P*-value was abbreviated in the figure because 12 significantly differences were detected. Please see Table S4. *P*-value shows the results of the post hoc test with Tukey’s adjustment method in a generalized linear mixed model (gamma distribution (**A** and **C**) and Poisson distribution (**B**), log link function).

We further examined which behavioral component could influence the latency to mate in naïve males in repeated mating tests. First, we compared the number of courtship displays (AIC (full) = 840.97, AIC (null) = 871.26, deviance = 820.97, deviance (null) = 865.26, Chisq = 44.29, Df = 7, Pr (> Chisq) = 1.88E−07) and the latency to the first courtship display (AIC (full) = 227.12, AIC (null) = 256.26, deviance = 205.12, deviance (null) = 248.26, Chisq = 43.14, Df = 7, Pr (> Chisq) = 3.13E−07) between naïve and experienced males, revealing that the latency to the first courtship display significantly decreased after the first mating experience in naïve males (Fig. 2A, Table S2). On the other hand, the number of courtship displays varied widely (AIC (full) = 840.97, AIC (null) = 871.26, deviance = 820.97, deviance (null) = 865.26, Chisq = 44.29, Df = 7, Pr (> Chisq) = 1.88E−07) in either naïve or experienced males (Fig. 2B, Table S4). Next, we compared the latency to mate after the first courtship display, which negatively correlated with the degree of female receptivity^17,21^, because females with high receptiveness tend to accept males immediately after the first courtship display. The latency was significantly decreased (AIC (full) = 365.53, AIC (null) = 384.91, deviance = 343.53, deviance (null) = 376.91, Chisq = 33.38, Df = 7, Pr (> Chisq) = 2.25E−05) in naïve males (Fig. 2C, Table S3), and strongly suggested that the female mating experience with the naïve males enhanced female receptivity. Furthermore, we analyzed the number of wrappings [(AIC (full) = 440.71, AIC (null) = 433.09, deviance = 420.72, deviance (null) = 427.09, Chisq = 6.37, Df = 7, Pr (> Chisq) = 0.497)] and the number of wrapping rejections [(AIC (full) = 313.55, AIC (null) = 318.683, deviance = 293.55, deviance (null) = 312.68, Chisq = 19.14, Df = 7, Pr (> Chisq) = 0.00777)], and these numbers varied widely in both naïve and experienced males (Fig. S1, Table S5). Next, we analyzed behavioral transition probability in the mating tests, and found no significant differences in any of the behavioral transitions (Fig. S2, Table S8). Accordingly, we revealed that the mating experience in naïve males shortened “the latency to the first courtship display” as well as “the latency to mate after the first courtship display”, suggesting that the mating experience in naïve males enhanced both male and female mating activities.

### The behavioral change in naïve males occurred only in fixed dyads

**Figure 3:**
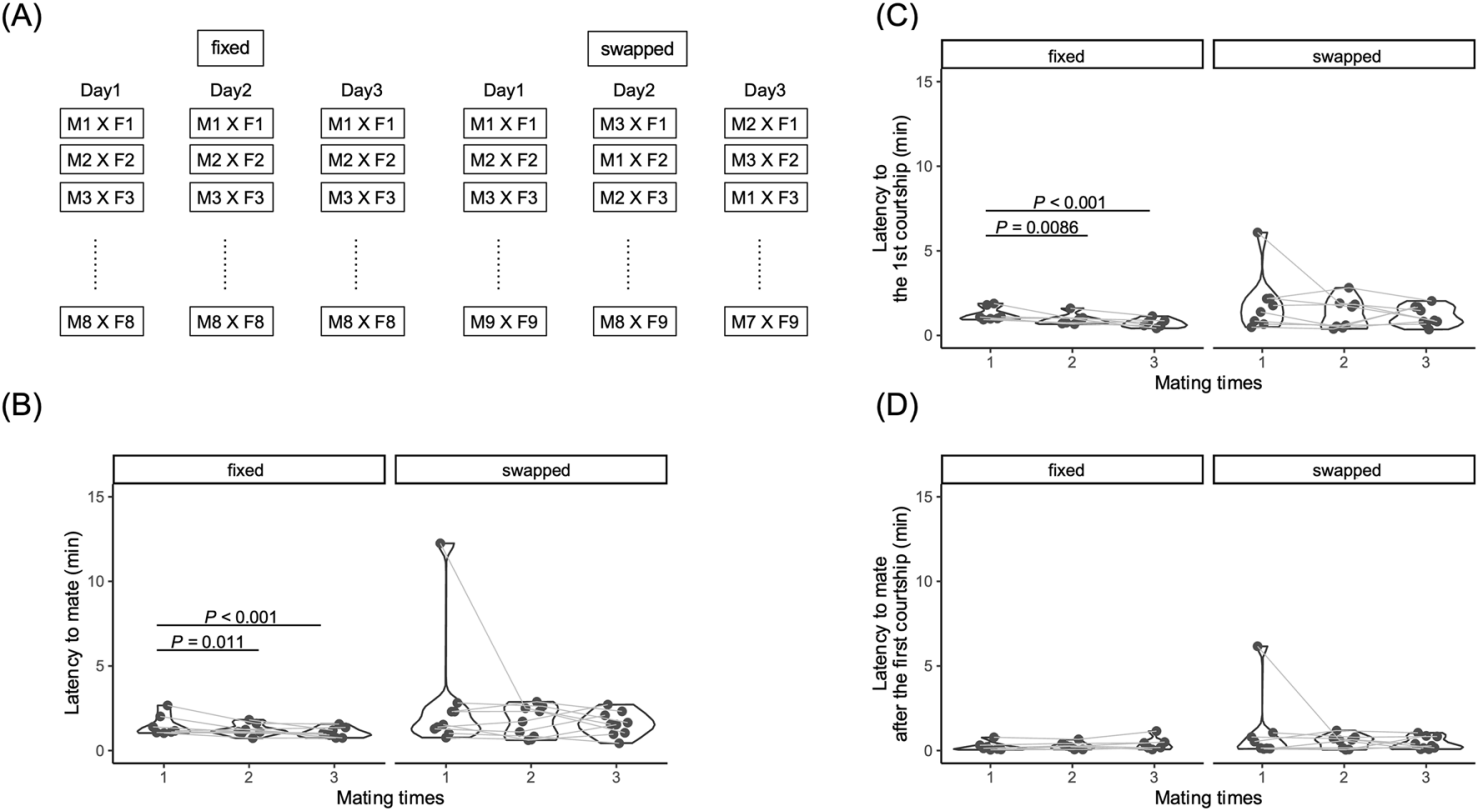
Effect of the familiarization (repeated matings with the same partner) on mating behavior of naïve males. (**A**) Outline of the behavioral experiment. Eight (fixed condition) and 9 (swapped condition) males were used for this study. (**B**) Latency to mate (left: fixed group, right: swapped group). The latency to mate significantly decreased only in the fixed group, and not in the swapped group. (**C**) Latency to the first courtship display (left: fixed group, right: swapped group). The latency significantly decreased only in the fixed group, and not in the swapped group. (**D**) Latency to mate from the first courtship. Each dot represents the results of each individual and the shapes show the experimental No. *P*-value shows the results of the post hoc test with Tukey’s adjustment method in a generalized linear mixed model (gamma distribution, log link function).

The first mating experience in naïve males mainly influenced the latency to mate and the latency to the first courtship display in fixed dyads in 7 continuous mating tests. Here we examined whether this effect was specific for fixed dyads. To compare mating behavior between fixed dyads and swapped dyads, we performed mating tests for 3 days (Fig. 3A, each n = 8). ‘Fixed’ dyads were those in which the male mates with the same female every day, while ‘swapped’ dyads were those in which the male mates with a different female each day. The latency to mate were significantly decreased only in the fixed group (AIC (full) = 453.39, AIC (null) = 468.10, deviance = 437.39, deviance (null) = 462.10, Chisq = 24.71, Df = 5, Pr (> Chisq) = 1.58E−04, Fig. 3B, Table S6). The latency to the first courtship display was also significantly decreased in the fixed group, but did not change in swapped dyads (AIC (full) = 45.48, AIC (null) = 62.57, deviance = 29.48, deviance (null) = 56.57, Chisq = 27.09, Df = 5, Pr (> Chisq) = 5.48E−05, Fig. 3C, Table S7). The latency to mate after the first courtship display; index of female receptivity (Fig. 3D, AIC (full) = − 11.34, AIC (null) = − 11.99, deviance = − 27.34, deviance (null) = − 17.99, Chisq = 9.35, Df = 5, Pr (> Chisq) = 0.0959), numbers of other events (Fig. S3, [courtships (AIC (full) = 177.50, AIC (null) = 170.56, deviance = 163.50, deviance (null) = 166.56, Chisq = 3.06, Df = 5, Pr (> Chisq) = 0.691)], [wrapping (AIC (full) = 148.86, AIC (null) = 140.99, deviance = 134.86, deviance (null) = 136.99, Chisq = 2.13, Df = 5, Pr (> Chisq) = 0.831)], [wrapping rejection (AIC (full) = 92.20, AIC (null) = 86.47, deviance = 78.20, deviance (null) = 82.48, Chisq = 4.27, Df = 5, Pr (> Chisq) = 0.511)]), and behavioral transitions (Fig. S4, Table S9) did not significantly change in either fixed or swapped dyads. The latency to mate and the latency to the first courtship display significantly decreased after the second encounter (day 3). These findings strongly suggest that the naïve males recognized the first mating partner and thus the latency to mate was decreased according to the mating experience.

### Mating experience in naïve males changed gene expression in the brain

To evaluate the effect of the mating experience in naïve males on the brain gene expression patterns, we compared gene expression profiles using whole brains between the naïve and post-naïve male medaka that had 2 mating experiences (Fig. S5, Table S10). In the present study, we performed RNA-seq analysis using the whole brain with the pituitary, because in some fish species such as zebrafish and cichlid, social status has significant effects on gene expression at the whole brain level^22,23^. We identified 10 differentially expressed genes (DEGs) that were upregulated by the mating experience and had a greater than twofold change, suggesting that the first mating experience could influence brain gene expression in male medaka. We listed the DEGs in descending order of the expression level (FPKM). Interestingly, we found that 3 genes (*tshba*, *dio2*, *klf9*) of the top 10 DEGs were associated with functional expression of the thyroid hormone system (Table 1). *tshba* encodes a thyroid-stimulating hormone that promotes the synthesis and secretion of inactivated thyroid hormone (T4). *dio2* encodes type II iodothyronine deiodinase, which converts inactivated thyroid hormone to activated thyroid hormone (T3). *klf9* encodes a transcription factor, Krüppel-like factor 9, which is induced by thyroid hormone (T3)^24^.

**Table 1:** RNA-seq results.

	Gene	log_2_|FC|	FDR	FPKM	FPKM
				(Naïve)	(Post-naïve)
1	*fkbp5*	1.89	1.010E−18	23.68	87.57
2	***tshba***	2.72	3.320E−39	6.99	45.93
3	*hapln2*	1.12	4.760E−05	19.22	41.74
4	LOC101155558	1.40	3.284E−03	11.44	30.26
5	*macrosialin*	1.04	1.990E−05	14.57	29.91
6	*stat1a*	1.13	5.290E−07	10.18	22.34
7	*lgals17*	1.76	1.730E−06	4.97	16.87
8	***dio2***	1.05	9.331E−04	7.30	15.10
9	*lgals3bp*	1.39	5.390E−07	5.77	15.10
10	***klf9***	1.10	4.730E−08	6.24	13.36

Genes that are related to the functional expression of the thyroid hormone system are in bold.

List of differentially expressed genes (DEGs) that were more highly expressed in post-naïve samples (2 matings) than in naïve samples. Genes are ordered from high to low expression using FPKM values as an index.

## Discussion

The findings of the present study revealed that naïve male medaka fish alter their mating behavior according to their first mating experience. Interestingly, the pattern of behavioral alterations after the first mating in medaka fish was quite different from those of other species such as fruit flies^2,25^, mice^3,4^, and rats^5^, in which the mating experience increases male sexual motivation, but does not influence mating activity toward familiar females. In medaka fish, mating experience with the same partners decreased the latency of naïve males to mate, but not experienced males. This finding suggests that naïve medaka males recognize the female that they mated with previously and mating activity with the familiar female is enhanced, while in experienced medaka males, mating activity is not enhanced for the familiar female. In addition, we observed the interesting phenomenon that female receptivity was enhanced only after they mated with naïve males. The mating experience of naïve males significantly decreased “the latency to the first courtship” as well as “the latency to mate after the first courtship. The index of the latency to the first courtship negatively correlated with male mating activity, while the latency to mate after the first courtship negatively correlated with female receptiveness. The previous study showed that females could be visually familiarized with proximally located males and that the visual familiarization enhanced female receptiveness toward the familiarized males^26^. If naïve males were in close proximity to the female more frequently than the experienced males, it could enhance female receptiveness via visual familiarization.

What is the adaptive significance of the mating experience-dependent change in behaviors? Why was mating activity with the same partner enhanced for naïve males and not for experienced males? Some possible explanations for the adaptive significance of these behaviors are as follows. One possibility is that the mating experience erases a certain behavioral property of juvenile individuals, which can recognize familiar individuals and tend to approach them to form a kin group^27^. Humbug damselfish^28^ and three-spot dascyllus^29^ juveniles tend to approach familiar individuals. Juvenile guppies tend to form kin groups to achieve effective transfer and protect against predators^27^. Juvenile cichlid fish maintained in a kin group grow faster^30^ and approach a predator for surveillance more often than solitary individuals that do not live in a group^31^. Further studies are needed to elucidate whether juvenile and naïve medaka fish tend to approach familiar mates to form kin groups like other fish species.

**Figure 4:**
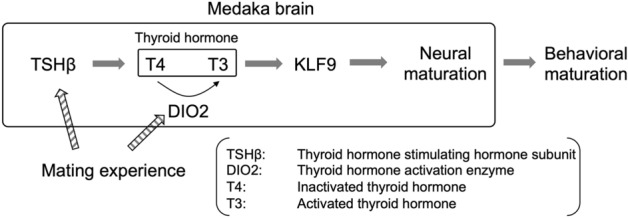
Possible model of the effect of the first mating experience in male medaka. In this study, we found that 3 genes (*tshba*, *dio2*, *klf9*) were upregulated after the first mating experience. *tshba* encodes thyroid stimulating hormone, which activates inactivated thyroid hormone (T4). DIO2, an enzyme that converts T4 to activated thyroid hormone T3. KLF9 is expressed depending on the T3 level and contributes to spine maturation. The first mating experience may lead to maturation of male mating behavior via neural maturation depending on the thyroid hormone system in the male medaka brain.

Various neurochemical substrates might be involved in experience-dependent plasticity in social/mating behavior, including oxytocin^32^, glutamate^33^, and opioids^34^. Among mammals and nonmammalian vertebrates, catecholamines such as dopamine (DA) and norepinephrine (NE) regulate the expression of sexual behaviors^35,36^. The findings of the present study suggest the mating experiences of naïve males induce the upregulation of 3 genes (*tshba*, *dio2*, *klf9*) related to functional expression of the thyroid hormone, and other genes such as *fkbp5 and hapln2*. In rodent brains, early-life stress exposure increases *fkbp5* expression in the brain and FKBP5 regulates glucocorticoid receptor activity^37^. High *fkbp5* expression and early-life stress interact to increase anxiety-like behavior mediated by AKT signaling in association with hippocampal synaptic plasticity^38^. Hapln2 (also called Bral1) is essential for the formation of the functional extracellular matrix and neuronal conductivity in mice^39^. More importantly, transcriptome analysis suggested the possible involvement of thyroid hormone in the effect of experience-dependent plasticity in mating behavior. Thyroid hormone is required for brain maturation and development^40,41^ as well as experience-dependent behavioral plasticity in chick imprinting^42^. In newly hatched (1-day-old) chicks, imprinting upregulates *dio2* gene expression, which is required for memory formation associated with imprinting^42^. The thyroid hormone is also involved in behavioral plasticity in mating behavior. In Japanese quail, thyroid hormone is a trigger hormone for the release of gonadotropin-releasing hormone in the brain to mature gonads for reproduction^43^. A relationship between seasonal reproduction and thyroid hormone in the brain is reported across species, including mice^44^ and fish^45^, among seasonally reproductive animals. In this study, sexually mature males were used for the mating test and there was no change of seasonal information (i.e., water temperature and day length remained the same) in the laboratory, but medaka do show seasonal reproductivity^46^, suggesting that the thyroid hormone system is activated in the brain by the mating experience and not a seasonal change. To our knowledge, there are no reports of thyroid hormone activation triggered by the mating experience in any species. In addition, *klf9,* which is a transcription factor dependent on the thyroid hormone^24^, contributes to dendritic spine maturation in the mouse hippocampus^47^. The mating experience shapes mating behavior and sexual motivation in association with morphological changes in the mPOA, the center of male mating behavior in rodents^48,49^. Therefore, the mating experience may shape male mating social behavior by changing the neuronal morphology according to thyroid hormone levels in male medaka (Fig. 4). The center of the mPOA, including the sexually dimorphic nucleus of the preoptic area, is more activated in naïve male rats than in experienced male rats^13^. In addition, knockdown of *vgf* expression erases the behavioral maturation dependent on the first mating experience^5^. Interestingly, thyroid hormone regulates *vgf* expression in hamsters^50^, implying that experience-dependent behavioral changes in male rodents are also related to the thyroid hormone system. These findings, however, are fragmented and further studies are necessary to elucidate whether the molecular mechanisms associated with male mating maturation dependent on the first mating experience are conserved among vertebrates.

## Supplementary Information

Supplementary Information.

## Data Availability

All sequence read data are available from DDBJ (DRA013480, https://ddbj.nig.ac.jp/resource/sra-submission/DRA013480) and the data that support the findings of this study are available from the corresponding author, [H. T.], upon reasonable request.
