# Supplementary material for: Mating experiences with the same partner enhanced mating activities of naïve male medaka fish
Source: Sci Rep. 2022 Nov 16;12:19665. doi: 10.1038/s41598-022-23871-w (PMC9668913; doi:10.1038/s41598-022-23871-w)
Supplement: Supplementary file 1 — Supplementary Information. [file 41598_2022_23871_MOESM1_ESM.docx]

Mating experiences with the same partner enhanced mating activities of naïve male medaka fish

Masahiro Daimon^1,2^, Takafumi Katsumura^2,3^, Hirotaka Sakamoto^2^, Satoshi Ansai^1^ and Hideaki Takeuchi^1,2,*^

^1^Graduate School of Life Sciences, Tohoku University, Sendai, Miyagi, 980-8577 Japan

^2^Graduate School of Natural Science and Technology, Okayama University, Okayama, Okayama 700-0082 Japan

^3^Department of Anatomy, Kitasato University School of Medicine, Sagamihara, Kanagawa, 252-0374 Japan

^*^hideaki.takeuchi.a8@tohoku.ac.jp


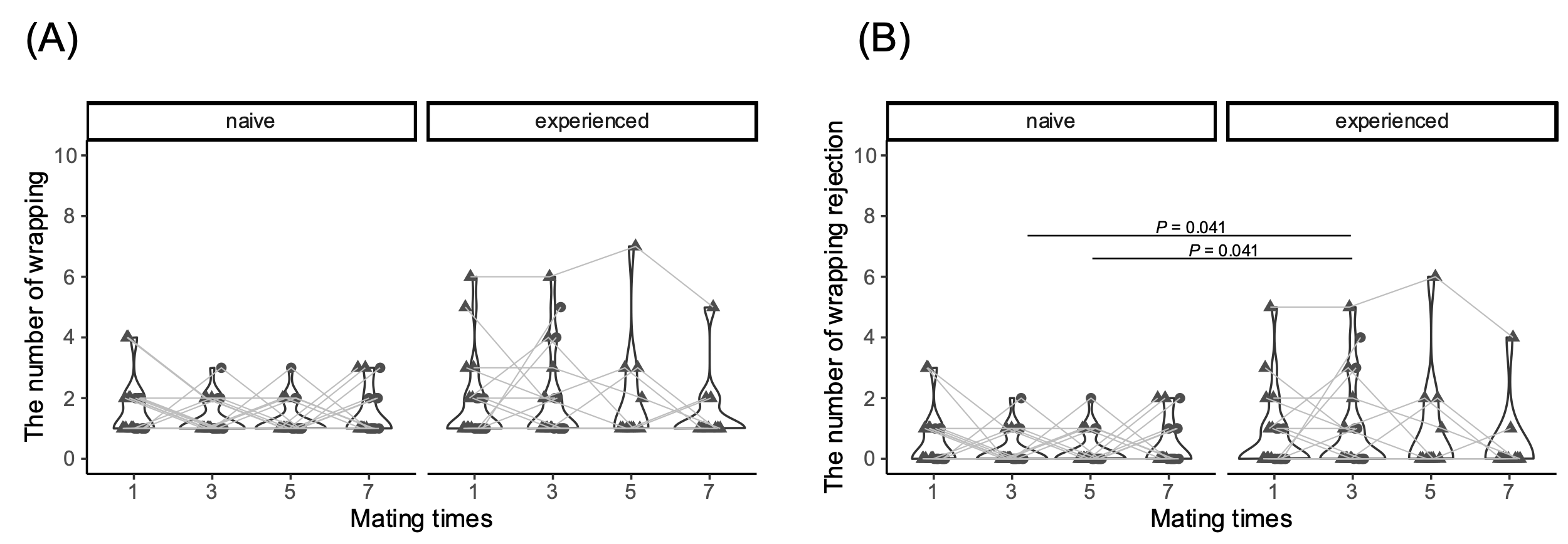


**Figure S1** The number of wrappings (A) and wrapping rejections (B). Each dots represents the results of each individual and shapes shows the experimental No (carried out 2 laboratories). There were no significantly differences whose *P*-value < 0.05 calculated by Tukey’s adjustment method in a ﻿generalized linear mixed model (poisson distribution, log link function).


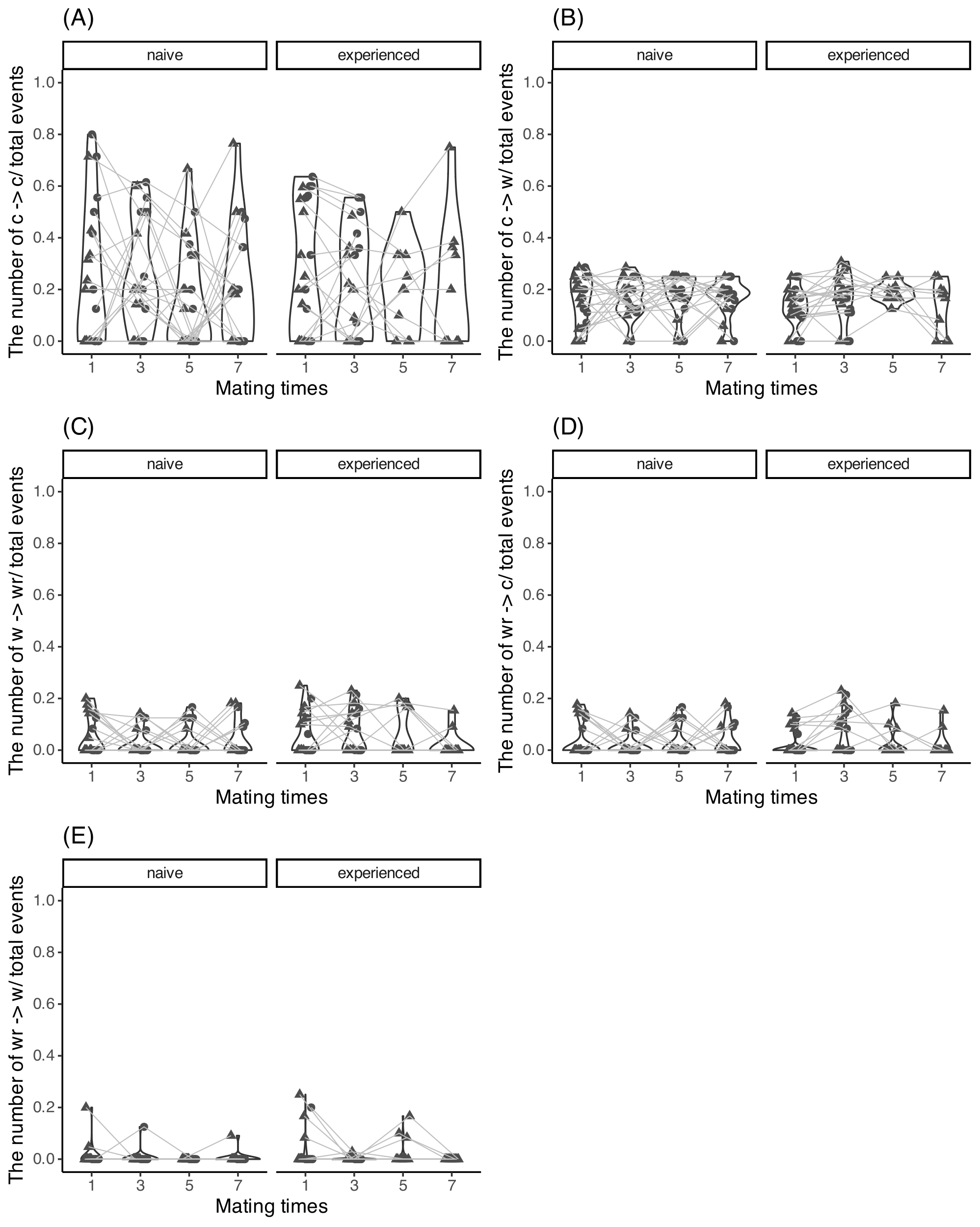


**Fig. S2** Behavioral transition probability of (A) courtship(c) -> courtship, (B) courtship -> wrapping (w), (C) wrapping -> wrapping rejection (wr), (D) wrapping rejection -> courtship, (E) wrapping rejection -> wrapping in mating test using swapped dyads. Each dots the results of each individual and shapes shows the experimental No (carried out 2 laboratories). There were no significantly differences whose P-value < 0.05. The way of statistical analysis are as same as in the Fig. S1.


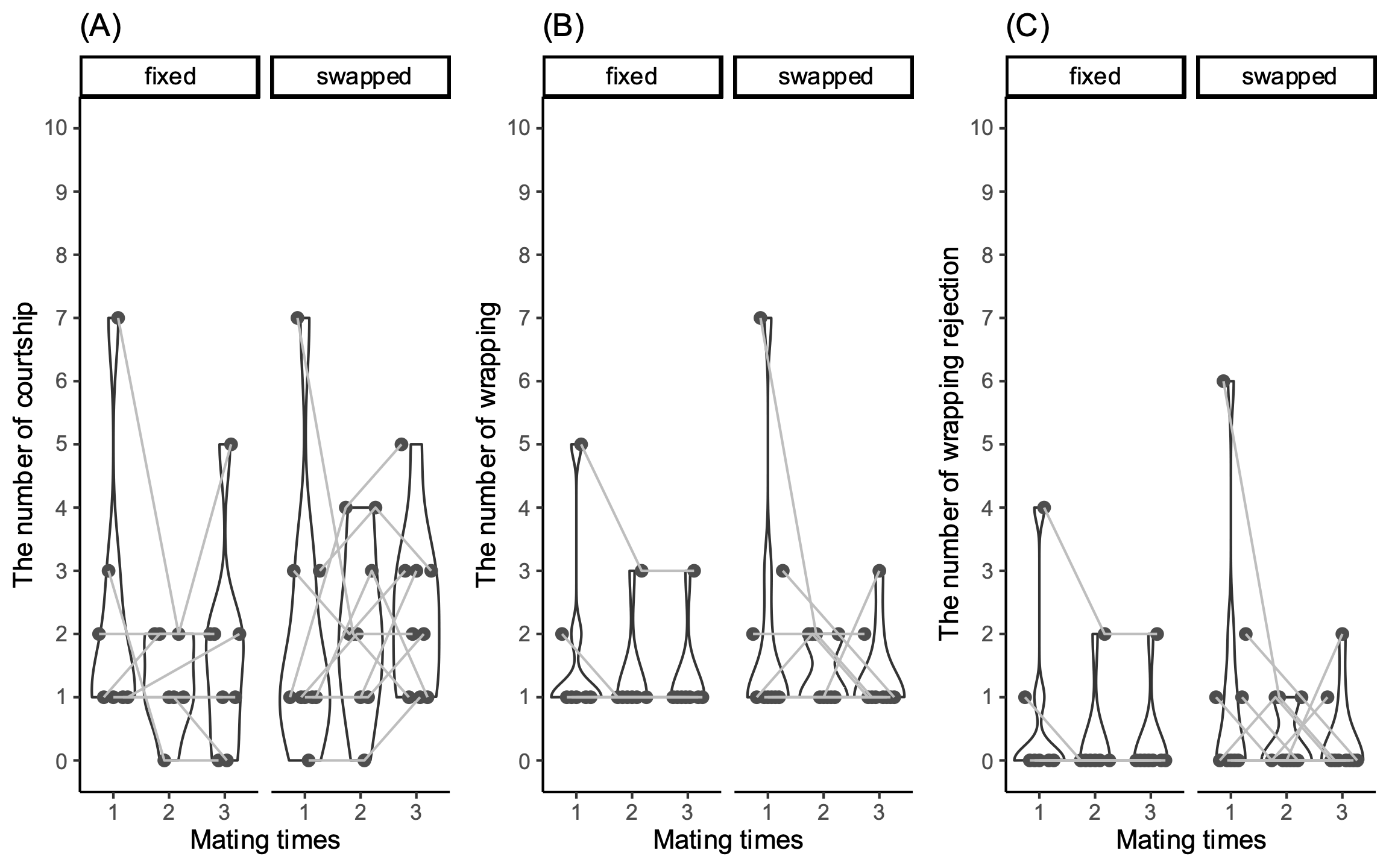


**Fig. S3** The number of courtship (A), wrapping (B) and wrapping rejection (C) in mating test using swapped dyads. Each dots the results of each. There were no significantly differences whose P-value < 0.05. The way of statistical analysis are as same as in the Fig. S1.


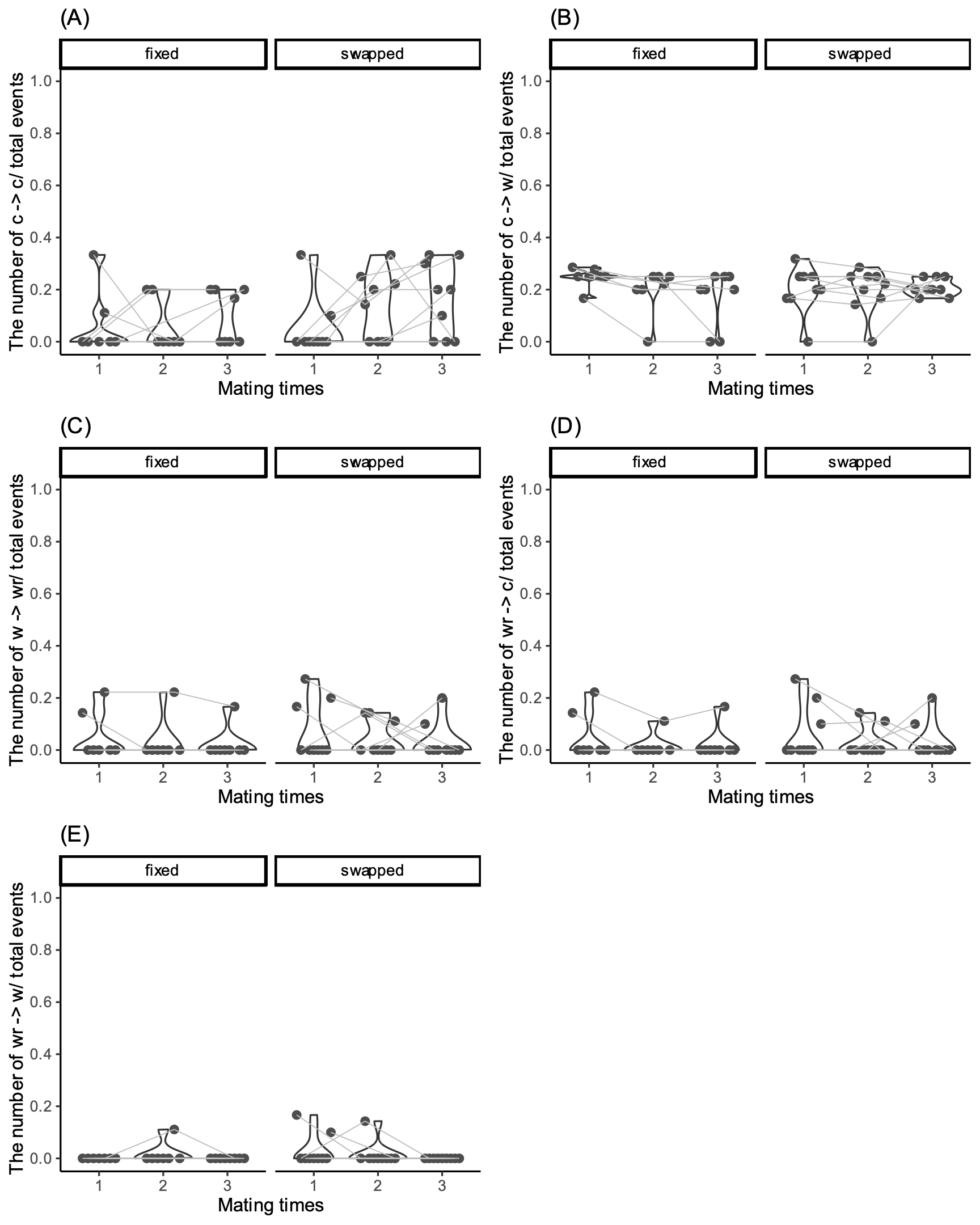


**Figure S4** The number of behavioral transition, (A) courtship (c) -> courtship (c), (B) courtship(c) -> wrapping (w) , (C) wrapping rejection (W.R) -> courtship, (D) wrapping -> wrapping rejection, (E) wrapping rejection -> wrapping in mating test using swapped dyads. Each dots the results of each individual and shapes shows the experimental No. There were no significantly differences whose P-value < 0.05. The way of statistical analysis are as same as in the Fig. S1.

**Figure S5** The time course of the extraction brain tissues and outline of the RNA-seq.

**Table S1** The result of the multiple comparison between latency to mate, mating times and types in the fixed group related to Fig. 1(C).

**Table S2** The result of the multiple comparison between latency to the first courtship, mating times and types in the fixed group related to Fig. 2(A).

**Table S3** The result of the multiple comparison between latency to mate after the first courtship, mating times and types in the fixed group related to Fig. 2(C)

**Table S4** The result of the multiple comparison between the number of courtships, mating times and types in the fixed group related to Fig. 2(B).

**Table S5** The result of the multiple comparison between the number of wrapping rejections, mating times and types in the fixed group related to Fig. S1(B).

**Table S6** The result of the multiple comparison between latency to mate, mating times and types in the swapped group related to Fig. 3(B).

**Table S7** The result of the multiple comparison between latency to the first courtship, mating times and types in the swapped group related to Fig. 3(C).

**Table S8** Results of the GLMM model selection of behavioral transition probability in the mating test using fixed groups. ∆AIC (AIC full model – AIC null model) and Pr (>Chisq) were evaluated to reveal the effect of the mating experience and mating times between naïve and experienced males.

**Table S9** Results of the GLMM model selection of behavioral transition probability in the mating test using swapped groups. ∆AIC (AIC full model – AIC null model) and Pr (>Chisq) were evaluated to reveal the effect of the mating experience and mating times between fixed and swapped conditions.

**Table S10** Sample information of the RNA-seq
